# Neural biomarker diagnosis and prediction to mild cognitive impairment and Alzheimer’s disease using EEG technology

**DOI:** 10.1186/s13195-023-01181-1

**Published:** 2023-02-10

**Authors:** Bin Jiao, Rihui Li, Hui Zhou, Kunqiang Qing, Hui Liu, Hefu Pan, Yanqin Lei, Wenjin Fu, Xiaoan Wang, Xuewen Xiao, Xixi Liu, Qijie Yang, Xinxin Liao, Yafang Zhou, Liangjuan Fang, Yanbin Dong, Yuanhao Yang, Haiyan Jiang, Sha Huang, Lu Shen

**Affiliations:** 1 Department of Neurology, Xiangya Hospital, Central South University, Changsha, China; 2 National Clinical Research Center for Geriatric Disorders, Central South University, Changsha, China; 3 Engineering Research Center of Hunan Province in Cognitive Impairment Disorders, Central South University, Changsha, China; 4 Hunan International Scientific and Technological Cooperation Base of Neurodegenerative and Neurogenetic Diseases, Changsha, China; 5 Key Laboratory of Hunan Province in Neurodegenerative Disorders, Central South University, Changsha, China; 6 Center for Interdisciplinary Brain Sciences Research, Department of Psychiatry and Behavioral Sciences, Stanford University School of Medicine, Stanford, CA USA; 7 Brainup Institute of Science and Technology, Chongqing, China; 8 Department of Geriatrics, Xiangya Hospital, Central South University, Changsha, China; 9 Mater Research Institute, The University of Queensland, Woolloongabba, Queensland 4102 Australia; 10 Key Laboratory of Organ Injury, Aging and Regenerative Medicine of Hunan Province, Changsha, China

**Keywords:** Mild cognitive impairment, Alzheimer’s disease, Electroencephalography, Diagnosis, Prediction, Biomarker

## Abstract

**Background:**

Electroencephalogram (EEG) has emerged as a non-invasive tool to detect the aberrant neuronal activity related to different stages of Alzheimer’s disease (AD). However, the effectiveness of EEG in the precise diagnosis and assessment of AD and its preclinical stage, amnestic mild cognitive impairment (MCI), has yet to be fully elucidated. In this study, we aimed to identify key EEG biomarkers that are effective in distinguishing patients at the early stage of AD and monitoring the progression of AD.

**Methods:**

A total of 890 participants, including 189 patients with MCI, 330 patients with AD, 125 patients with other dementias (frontotemporal dementia, dementia with Lewy bodies, and vascular cognitive impairment), and 246 healthy controls (HC) were enrolled. Biomarkers were extracted from resting-state EEG recordings for a three-level classification of HC, MCI, and AD. The optimal EEG biomarkers were then identified based on the classification performance. Random forest regression was used to train a series of models by combining participants’ EEG biomarkers, demographic information (i.e., sex, age), CSF biomarkers, and *APOE* phenotype for assessing the disease progression and individual’s cognitive function.

**Results:**

The identified EEG biomarkers achieved over 70% accuracy in the three-level classification of HC, MCI, and AD. Among all six groups, the most prominent effects of AD-linked neurodegeneration on EEG metrics were localized at parieto-occipital regions. In the cross-validation predictive analyses, the optimal EEG features were more effective than the CSF + *APOE* biomarkers in predicting the age of onset and disease course, whereas the combination of EEG + CSF + *APOE* measures achieved the best performance for all targets of prediction.

**Conclusions:**

Our study indicates that EEG can be used as a useful screening tool for the diagnosis and disease progression evaluation of MCI and AD.

**Supplementary Information:**

The online version contains supplementary material available at 10.1186/s13195-023-01181-1.

## Background

Alzheimer’s disease (AD) is the leading cause of dementia, accounting for an estimated 60-80% of cases worldwide [1]. Currently, there is no effective treatment for AD, and only very limited medications show the potentials for delaying the progression of this neurodegenerative disease at its early stage. On the other hand, amnestic mild cognitive impairment (MCI) is characterized by cognitive decline greater than normal for a person’s age and education level without notably interfering with activities of daily life [2]. It is well-accepted that MCI is a high-risk factor for the development of AD and reflects a prodromal predementia state of AD, with an estimated conversion rate of 10–15% per year [3]. Taken together, early diagnosis of AD, including detection at MCI or even early stage, may substantially benefit patients from disease-modifying treatments.

The gold standard of AD diagnosis is the Amyloid/Tau/Neurodegeneration (ATN) framework proposed by the National Institute on Aging and Alzheimer’s Association in 2018 [4]. In the ATN framework, the biological state of AD is classified through the identification of three biomarkers (i.e., amyloid, tau, and neurodegeneration) measured from cerebrospinal fluid (CSF) and positron emission tomography (PET) imaging [4]. Specifically, “A” represents the cortical amyloid PET ligand binding or low CSF Aβ42, “T” refers to elevated CSF phosphorylated tau (P-tau) and cortical tau PET ligand binding, and “N” indicates CSF T-tau, FDG PET hypometabolism, and atrophy on MRI [4]. The ATN framework has been shown to be a feasible way for AD diagnosis and can be also extended to quantify personalized risk profiling for individuals with MCI [5, 6]. However, this approach is typically conducted through a lumbar puncture or PET examination, which is costly, invasive, and highly relies on clinical infrastructure, thus significantly limiting its availability in clinical practice. As such, there are growing demands for the development of new approaches to aid the early diagnosis of AD.

Electroencephalography (EEG), a low-cost, non-invasive, and portable technique that directly measures neural activity with a high temporal resolution, has emerged as a potential tool for detecting neural biomarkers related to MCI and AD [7–10]. Numerous lines of evidence have validated the possibility of using EEG to distinguish MCI and AD patients from healthy cohorts with diverse sensitivity and specificity [11]. Overall, previous EEG studies involving both MCI and AD patients reported relatively consistent neural alterations compared to healthy cohort, including decreased alpha and beta rhythms activity and increased delta and theta oscillations, which are probably the most promising neural biomarkers for early detection of AD, due to their good correlations with patients’ cognitive function [11–15]. In addition, reduced complexity and coherence in EEG recordings, as well as decreased ratios of theta/gamma and high alpha/low alpha, were also reported as potential biomarkers for the diagnosis of AD [15–19].

Despite the convergence of evidence showing EEG-derived biomarkers linked to MCI and AD, there remain important challenges in bringing these findings into clinical practice. In particular, previous studies were largely conducted based on limited sample sizes. During the last three decades, as pointed out in a recent study, more than 95% of the studies that focused on EEG-based classification of MCI or AD were conducted with fewer than 100 participants, making relevant findings unconvincing [15]. Moreover, while AD is the most common form of dementia, symptoms of preclinical and early AD mostly overlap with other types of dementia such as frontotemporal dementia (FTD), dementia with Lewy bodies (DLB), and vascular cognitive impairment (VCI) [20–22]. However, most previous studies have commonly examined EEG signatures related to MCI and AD without the inclusion of other dementias. We argue that the sensitivity and specificity of such EEG biomarkers in early AD diagnosis should be further thoroughly validated on datasets with more types of dementia. Lastly, though a number of EEG biomarkers (e.g., power spectrum, entropy) have been extensively investigated in previous studies [23, 24], clear knowledge gaps remain regarding the effectiveness of such EEG biomarkers in the diagnosis and prediction of the progress of AD.

To address the aforementioned challenges, this study sought to examine key EEG biomarkers that are capable of distinguishing patients with MCI, AD, and other neurodegenerative dementias from healthy participants. We also aimed to investigate how the identified EEG biomarkers were associated with individual cognitive decline and CSF biomarkers. In addition, we implemented a machine learning approach and validated the added value of EEG biomarkers in assessing patients’ cognitive function (i.e., MMSE and MoCA), age of disease onset (ADO), and course of disease (COD).

## Materials and methods

### Participants

A total of 890 individuals were utilized in the study, including 189 patients with amnestic MCI, 330 patients with AD, 47 patients with FTD, 57 patients with VCI, 21 patients with DLB, and 246 healthy controls (HC). All patients were enrolled from the Department of Neurology, Xiangya Hospital, Central South University, between March 2017 and January 2022. The patients of MCI, probable AD, FTD, VCI, and DLB were diagnosed according to the respective clinical-based criteria [25–30]. All HC were recruited from Xiangya Health Management Center, who were matched with age and sex and reported without cognitive decline. The protocol was approved by the Institutional Review Board of Xiangya Hospital, Central South University. All participants or guardians signed the informed consent before the study.

### *APOE* genotyping

The gDNA was extracted from peripheral blood using the standard phenol-chloroform extraction method. All gDNA samples were diluted to 50 ng/μl. A 581-bp fragment was amplified using the following primers: forward 5-CCTACAAATCGGAACTGG-3, and reverse 5-CTCGAACCAGCTCTTGAG-3. Polymerase chain reaction (PCR) was performed as previously described [31]. Each PCR product was sequenced using an ABI 3730xl DNA analyzer (ABI, Louis, MO, USA).

### CSF collection and analysis

The CSF was collected through a lumbar puncture. The samples were then centrifuged at 2000× g and 4 °C for 10 min and stored at the temperature of −80 °C. All assessments of CSF biomarkers were measured using an enzyme-linked immunosorbent assay (ELISA), including beta-amyloid (1–40) (EQ 6511–9601), beta-amyloid (1–42) (EQ 6511–9601), total-tau (EQ 6531–9601), and pTau (181) (EQ 6591–9601-L) (EUROIMMUN, Germany). Four core biomarkers, including Aβ42, Aβ40, t-tau, and p-tau were then obtained. All procedures were performed in accordance with the manufacturer’s instructions. Briefly, samples were added to the reagent wells, and the plate was incubated for 3 h at 22°C ± 2°C. After washing, horseradish peroxidase solution was added and incubated for 90 min at 22°C ± 2°C. The plate was then washed with the provided washing buffer, and substrate solution was added. After 30 min incubation protected from light, stop-solution was added, and the optical density (OD) was measured using a microplate reader (Thermo, Waltham, MA, USA), at 450 nm, corrected by the reference OD at 620 nm within 30 min of adding the stop solution. Two technical replicates were performed on samples and standards, and the mean of the replicates was used for the final analysis.

### EEG collection and preprocessing

**Fig. 1:**
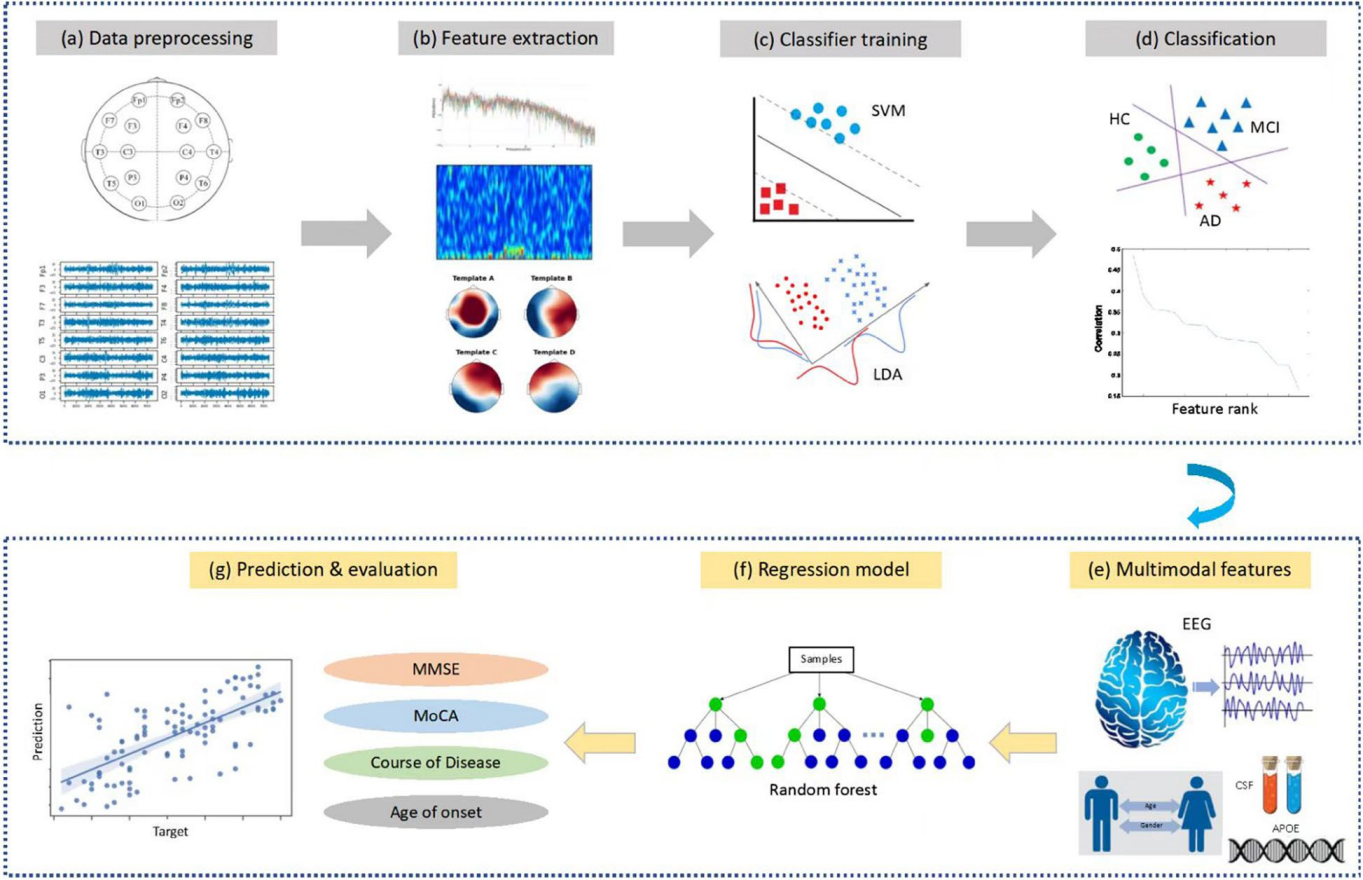
The schematic diagram of the classification **a**–**d** of HC/MCI/AD participants and assessment **e**–**g** of participants’ cognitive function and disease progression

EEG signal was recorded at 200 Hz from the participants in a 10-min eye-closed resting state. Participants were required to remain awake during the entire recording. Standard 16-channels montage was utilized according to the 10–20 International System (channels: Fp1, Fp2, F3, F4, F7, F8, T3, T4, T5, T6, C3, C4, P3, P4, O1, O2). All data analysis was performed using customized Python scripts (Python 3.9, Delaware, USA). The pipeline of the proposed classification and assessment framework is shown in Fig. 1. Briefly, the collected EEG signals were first band-pass filtered between 1 Hz and 55 Hz and further filtered by a 50-Hz notch filter to remove the powerline interference. The filtered data were then re-referenced using a common average re-reference approach. After that, all EEG traces were inspected using a 25-s sliding time window for motion artifact (e.g., spike) detection. Windowed data containing more than 30% artifacts were excluded, leaving the average length of EEG data at 6.2 min. As recommended by a guideline published recently [32], we then segmented the EEG data into a series of epochs using a 5-s time window (i.e., 1000 sample points), resulting in data size of M epochs Í 16 channelsÍ1000 points for each participant. A total of 38,925 epochs were finally obtained from all participants (HC, MCI, and AD).

### EEG analyses and feature extraction

We computed multiple types of EEG features, including absolute power, relative power, Hjorth metrics (activity, mobility, and complexity), time-frequency property (STFT), sample entropy, and microstate measures (lifetime, occurrence rate, converting rate). Before feature calculation, each 5s epoch data was filtered by a band-pass filter to extract several frequency components including delta (1–4 Hz), theta (4–8 Hz), alpha (8–13 Hz), beta (13–30 Hz), and gamma (30–45 Hz). We then computed all features from each frequency component of each epoch. Specifically, the absolute power and relative power features were extracted from each frequency band of each channel, while the Hjorth metrics and sample entropy were computed for each channel. Time-frequency (STFT) features were calculated from each frequency band across all channels, while microstate features were extracted across all channels. A detailed definition of these features can be found in the Supplementary material.

### Classification of HC, MCI, and AD based on EEG features

We performed a two-step three-level classification of HC, MCI, and AD groups using linear discriminant analysis (LDA) and support vector machine (SVM). Briefly, we first calculated the Pearson’s correlation coefficient between each individual feature dimension and all class labels (HC = 1, MCI = 2, AD = 3), resulting in a series of correlation coefficients between each EEG feature and the class labels. All EEG features were then sorted based on their absolute correlation coefficients in a descending manner, in which the features that yielded a higher coefficient represented more important features and were given higher priority in the classification of the three groups. With the sorted feature set, we started the classification using the feature with the highest priority, and then iteratively added other features one by one (in a forward manner) and evaluated the classification performance at each iteration. The optimal feature set was defined as the set that yielded the highest accuracy, representing the key EEG features essentially related to the diagnosis of MCI and AD. We randomly selected 80% of the samples as a training set, and the left 20% of the samples as a testing set. To avoid the overfitting problem, classifiers were trained using 5-fold cross-validation within the training set, in which 80% of the training set was used to train the model and 20% of the training set was used as the validating set.

### Statistical analyses

Among the optimal EEG features identified via the three-way classification, we further explored whether these EEG features could be used to distinguish MCI from AD groups as well as other subtypes of dementia (i.e., DLB, FTD, and VCI) through a series of ANCOVA tests that included age as a covariate. Significant EEG features were determined (*p* < 0.05) and tested for the between-group difference using a post hoc Tukey test (corrected for age, multiple comparisons were corrected using the FDR Benjamini-Hochberg method). To assess the association among individual neural activity, CSF, and cognitive function, we computed Pearson’s correlation between each EEG feature and the Mini-Mental State Examination (MMSE), Montreal Cognitive Assessment (MoCA) scores, and each CSF biomarker within the combined MCI-AD group.

### Assessment of cognitive decline in MCI and AD patients

Next, we aimed to assess how well the selected EEG-based biomarkers, demographic information (i.e., sex, age), the CSF biomarkers, and *APOE* phenotype can be used to assess individual cognitive function and disease profiles within the MCI and AD groups, through a machine-learning-based approach. Three types of feature sets, including EEG feature-only (EEG), CSF/*APOE* measures-only (CSF/*APOE*), and hybrid (EEG + CSF/*APOE* + demographic information), were independently selected to train a series of regression models for the prediction of the absolute scores of MMSE, MoCA, ADO, and COD, respectively. As the total number of patients with MCI and AD decreased due to missing measurements of CSF and *APOE*, random forest regression was used in the predictive analysis to avoid an overfitting problem [33]. Random forest regression is an ensemble technique capable of performing regression-based predictive analysis with the use of multiple binary decision trees. The basic idea behind this is to combine multiple simple predictive models in determining the final output rather than relying on a simple regression model. Here, we adopted a 10-fold cross-validation approach to perform relatively robust predictions of measures of interest. In each fold, we randomly split the available participants (MCI + AD) into a training set (80%) and a validating set (20%). After ten iterations, we combined all training and validating sets from each fold to form the complete training set and validating set for the prediction, respectively. The prediction performance of the regression models was assessed using two metrics. The first metric is the coefficient of determination (*R*^2^), a statistical measure that assesses how well the predicted scores approximate the target score. The coefficient of determination (*R*^2^) is calculated as:

$${R}^2=\textrm{MSS}/\textrm{TSS},$$

where MSS is the model sum of squares, which is the sum of the squares of the predicted variable minus the mean of the true target variable; TSS is the total sum of squares associated with the target variable, which is the sum of the squares of the target variable minus their mean. *R*^2^ ranges from 0 to 1, and a higher *R*^2^ indicates better goodness of fit for the observations.

The second metric is the mean absolute error (MAE) which measures the average magnitude of the errors in a set of predictions. MAE can be calculated as:

$$\textrm{MAE}=\frac{1}{n}\sum\nolimits_{i=1}^n\left({y}_i-\hat{y_i}\right),$$

where n is the number of samples, *y*_*i*_ is the target score of the *i*th sample, and $\hat{y_i}$ is the predicted score of the *i*th sample.

## Results

### Demographic information

The demographic information of 890 participants is summarized in Table 1. No significant difference in age and sex distribution were observed among the MCI, AD, and HC participants (*p* = 0.50 for age; *p* = 0.29 for sex).

**Table 1:** Demographic and clinical characteristics of six groups of individuals

	MCI (*n*=189)	AD (*n*=330)	VCI (*n*=57)	FTD (*n*=47)	DLB (*n*=21)	HC (*n*=246)
Age (years)	64.77 ± 9.30	64.60 ± 9.75	67.18 ± 9.80	61.36 ± 8.69	72.01 ± 9.07	63.85 ± 8.20
Sex (male, %)	68, 35.98	121, 36.67	31, 54.39	23, 48.94	13, 61.90	104, 42.28
ADO	62.78 ± 9.42	61.80 ± 9.91	61.47 ± 11.06	61.74 ± 10.42	62.67 ± 10.49	-
COD	1.98 ± 1.91	2.90 ± 2.49	1.75 ± 1.97	2.79 ± 1.65	2.00 ± 2.31	-
MMSE	22.70 ± 4.48	11.63 ± 6.28	13.51 ± 6.98	12.20 ± 7.41	12.76 ± 6.44	28.62 ± 1.09
MoCA	16.22 ± 5.01	7.02 ± 4.87	7.74 ± 4.86	7.68 ± 6.74	5.94 ± 5.65	-
Aβ42 (pg/ml)	587.46 ± 439.59	408.29 ± 217.28	-	-	-	-
Aβ40 (pg/ml)	8572.50 ± 6108.20	8286.91 ± 6000.22	-	-	-	-
Aβ42/40	0.09 ± 0.07	0.06 ± 0.04	-	-	-	-
t-tau (pg/ml)	316.58 ± 289.84	506.21 ± 316.95	-	-	-	-
p-tau (pg/ml)	76.79 ± 51.92	104.39 ± 51.94	-	-	-	-
*APOE ε4* (%)	43.86 (25/57)	52.00 (78/150)				

Fifty-seven with MCI and 150 with AD, completed *APOE* genotype testing, 43.86% and 52.00% of whom were *APOE ε4* carriers (at least one *ε4* allele), respectively. In addition, 28 with MCI and 87 with AD completed the CSF core biomarkers testing

*ADO* age of disease onset, *COD* course of disease

### EEG-based classification results

**Fig. 2:**
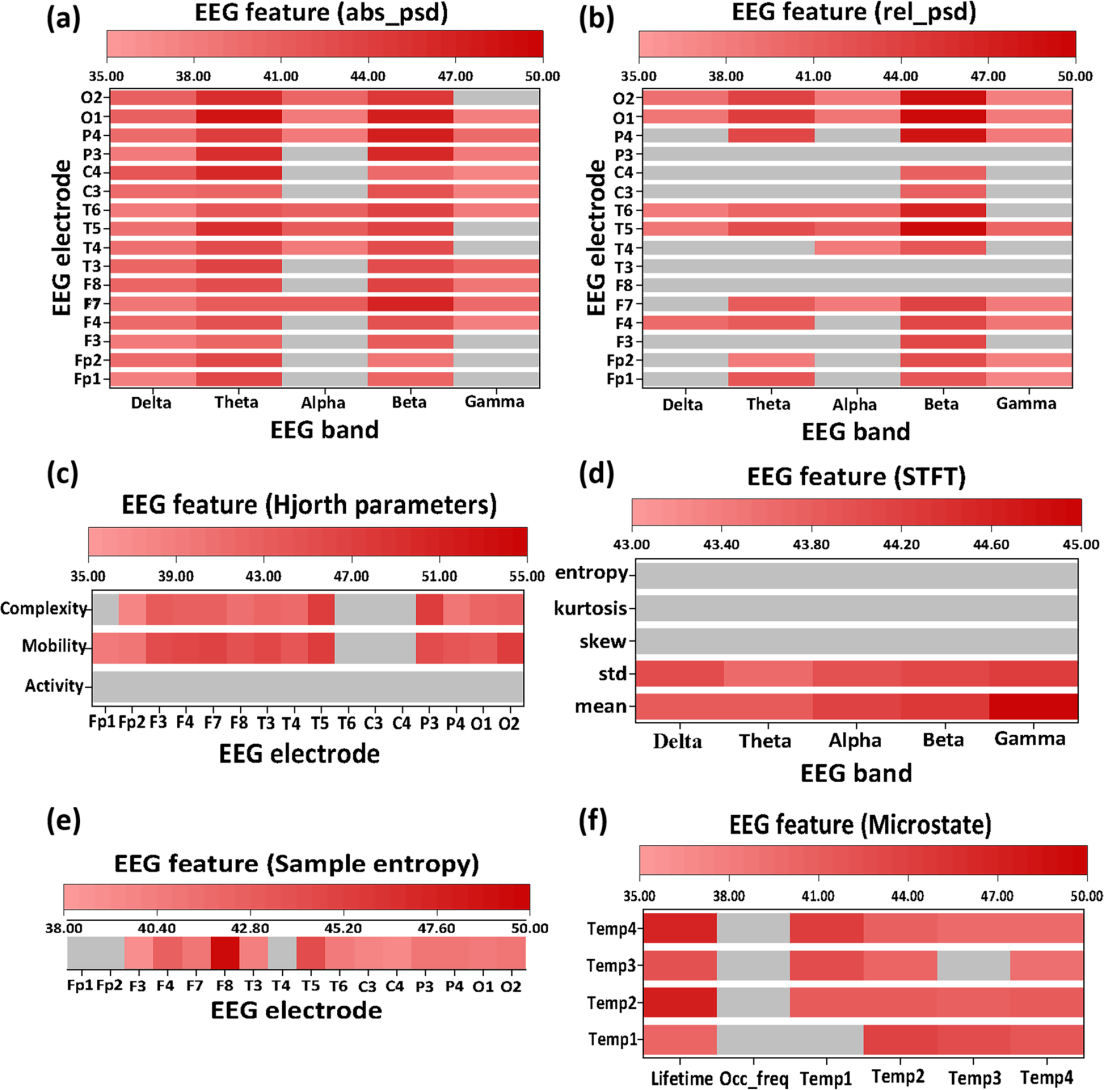
Distribution of the optimal feature set among all extracted EEG features in classification (indicated by red). Six types of EEG features were extracted, including **a** absolute PSD, **b** relative PSD, **c** Hjorth metrics (activity, mobility, and complexity), **d** time-frequency measures (STFT), **e** sample entropy, and **f** microstate measures (lifetime, occurrence rate, converting rate)

We identified 178 EEG features as the key features for the classification of HC, MCI, and AD. The optimal feature set covered partial features from each EEG feature category, particularly the absolute PSD and the complexity of EEG signals. The distribution of the optimal feature set among all extracted EEG features is shown in Fig. 2.

Table 2 summarizes the classification performance of binary classification and three-level classification. The metrics used to evaluate the classification performance included recall, precision, F1-score, and accuracy, given as:

1 $$\textrm{Recall}\ \left(\textrm{RC}\right)=\frac{\textrm{TP}}{\textrm{TP}+\textrm{FN}}$$

2 $$\textrm{Precision}\ \left(\textrm{PC}\right)=\frac{\textrm{TP}}{\textrm{TP}+\textrm{FP}}$$

3 $$\textrm{F}1\ \textrm{score}=2\ast \frac{\textrm{PC}\ast \textrm{RC}}{\textrm{PC}+\textrm{RC}}$$

4 $$\textrm{Accuracy}=\frac{\textrm{TP}+\textrm{TN}}{\textrm{TP}+\textrm{TN}+\textrm{FP}+\textrm{FN}}$$

**Table 2:** Classification performance of binary classification and triple classification using EEG-based features

Classifiers	Recall	Precision	F1 score	Accuracy
**HC vs. MCI**				
SVM	79.6%	75.8%	77.7%	76.7%
LDA	83.8%	78.1%	80.9%	79.8%
**HC vs. AD**				
SVM	86.4%	83.6%	85.0%	84.4%
LDA	84.7%	87.0%	85.8%	85.8%
**HC vs. MCI vs. AD**				
SVM	70.2%	70.7%	69.8%	70.2%
LDA	70.0%	70.3%	69.4%	70.0%

where TP, TN, FP, and FN represent the true positive, true negative, false positive, and false negative, respectively. Generally, *Recall* measures the model’s ability to correctly predict the positives out of actual positives (i.e., patients). A lower recall score would mean that some patients who are positive are termed as falsely negative. *Precision* measures the proportion of positively predicted labels that are actually correct. A low precision score would mean that some people who are negative are termed as falsely positive. *F1 score* represents the model score as a harmonic mean of precision and recall score, which is often used to provide high-level information about the model’s output quality. Model *accuracy* is defined as the ratio of true positives and true negatives to all positive and negative observations. That is, accuracy evaluates a model’s ability to correctly output a diagnosis out of the total predictions it made.

As shown in Table 2, the binary classification of HC vs. MCI achieved an accuracy of approximately 80% and an F1 score of 80.9%, while the binary classification of HC and AD achieved an accuracy of approximately 85% and an accuracy of 80%. For a three-level classification of HC vs. MCI vs. AD, both the accuracy and F1 score reached about 70%. Regardless of classification type, the SVM model showed similar classification performance compared to the LDA model.

### Comparison among MCI/AD and subtypes of dementia

**Fig. 3:**
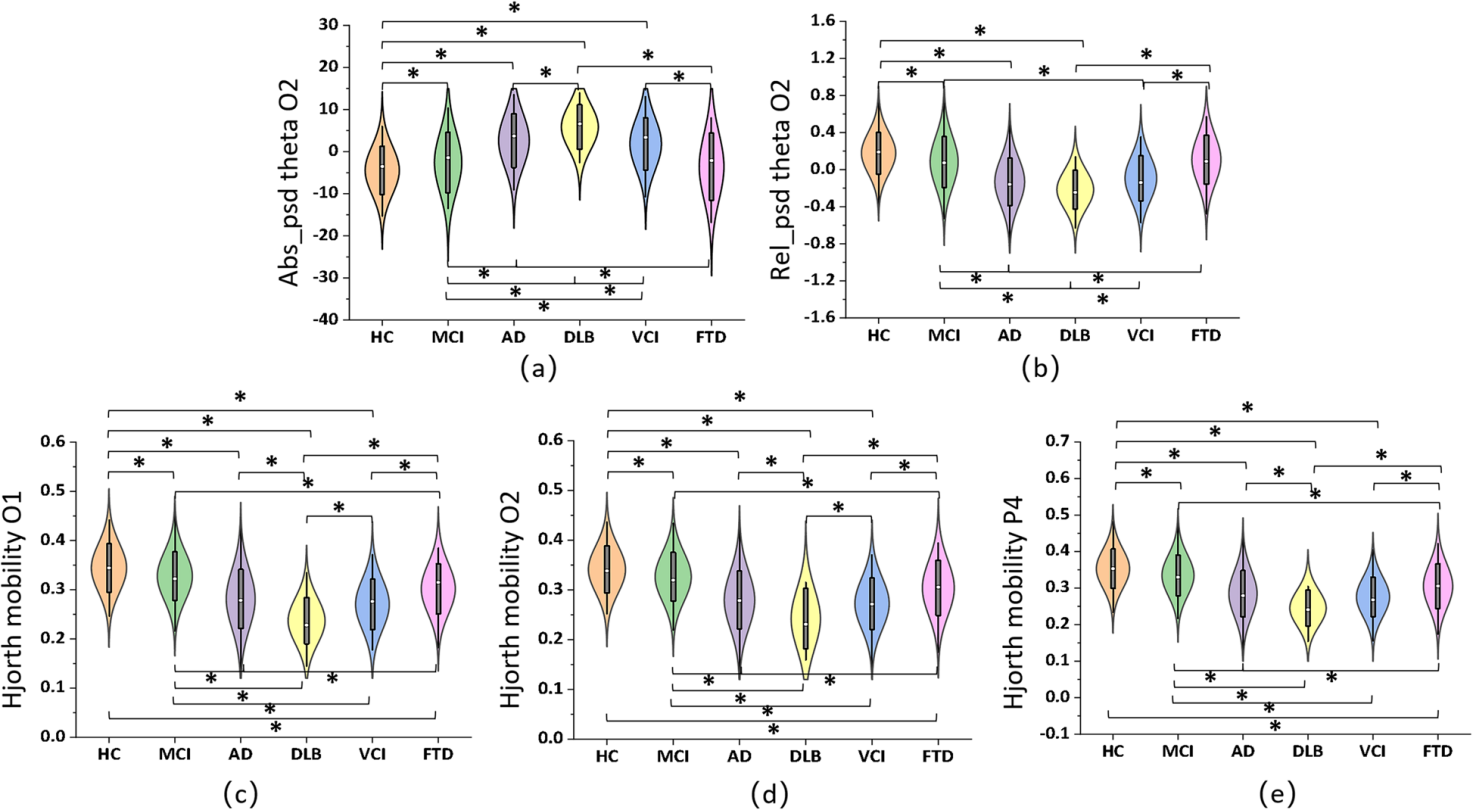
The key EEG biomarkers at parieto-occipital regions effectively recognized distinct neural patterns among six groups. Selected EEG features included **a** absolute theta PSD at O2 (*F* = 42.46, *p* < 0.001), **b** relative theta PSD at O2 (*F* = 50.11, *p* < 0.001), **c** Hjorth mobility at O1 (*F* = 51.08, *p* < 0.001), **d** Hjorth mobility at O2 (*F* = 50.14, *p* < 0.001), and **e** Hjorth mobility at P4 (*F* = 47.09, *p* < 0.001). “*” indicates that there is a significant between-group difference (*p*<0.05, FDR corrected)

Through a series of ANCOVA tests, we found significant differences among MCI, AD, and other subtypes of dementia in a total of 178 EEG features. Figure 3 displays five EEG features that showed representatively significant differences among all six groups (all *Fs* > 40, all *ps* < 0.0001), with results of the post hoc pair-wise comparisons (FDR corrected). Most of the selected features showed significant between-group differences, particularly among HC, MCI, and AD groups. These features mainly included the absolute theta PSD, relative theta PSD at the occipital area, and Hjorth mobility at the parieto-occipital area. Moreover, significant differences between MCI/AD groups and other subtypes of dementia, such as DLB, FTD, or VCI, were also observed in the selected EEG features.

### Brain-cognition-CSF relationship in patients with MCI and AD

**Fig. 4:**
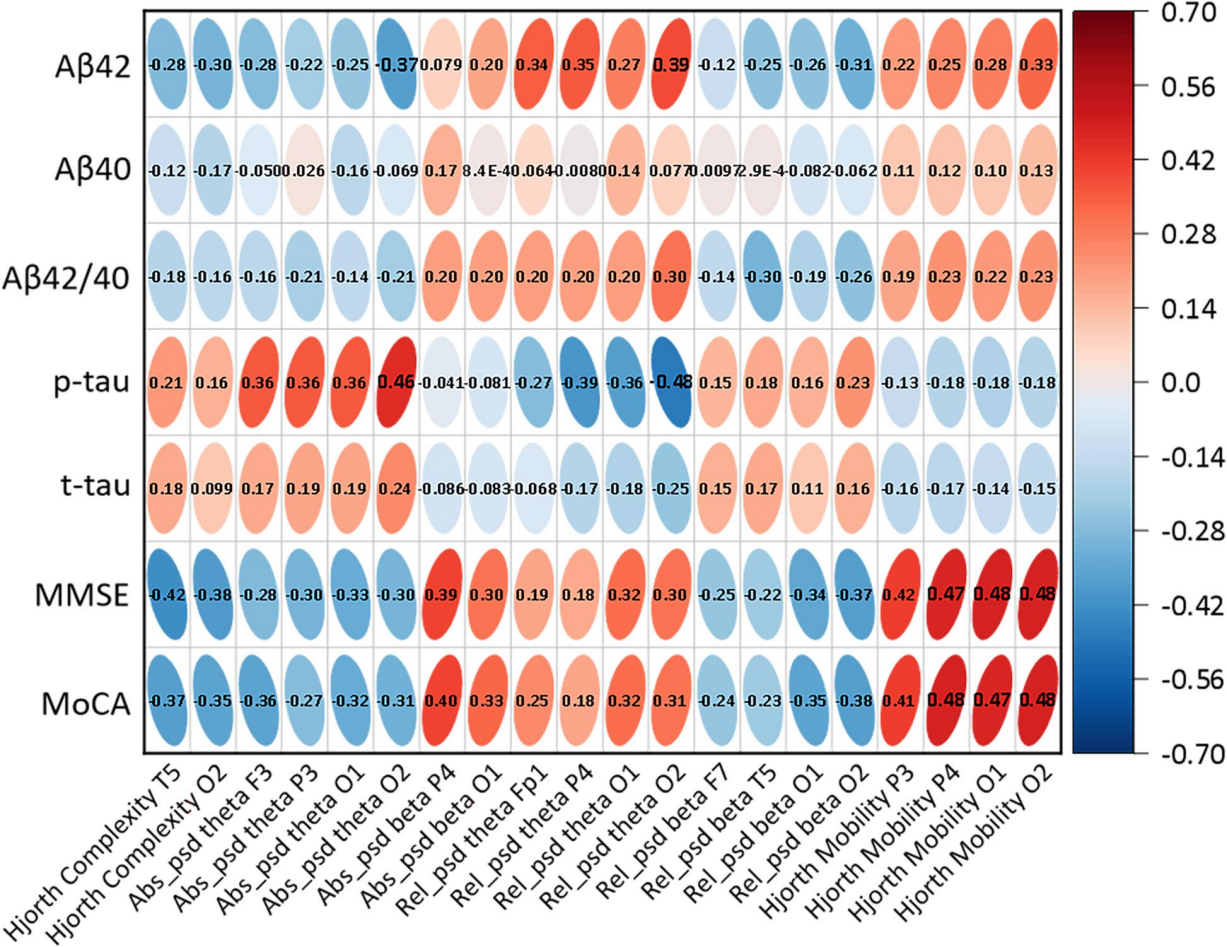
The correlational analyses among brain measures (EEG features), cognitive decline (MMSE, MoCA), and CSF biomarkers. Numbers within the Ellipses represent the correlation coefficients of all x–y pairings

**Fig. 5:**
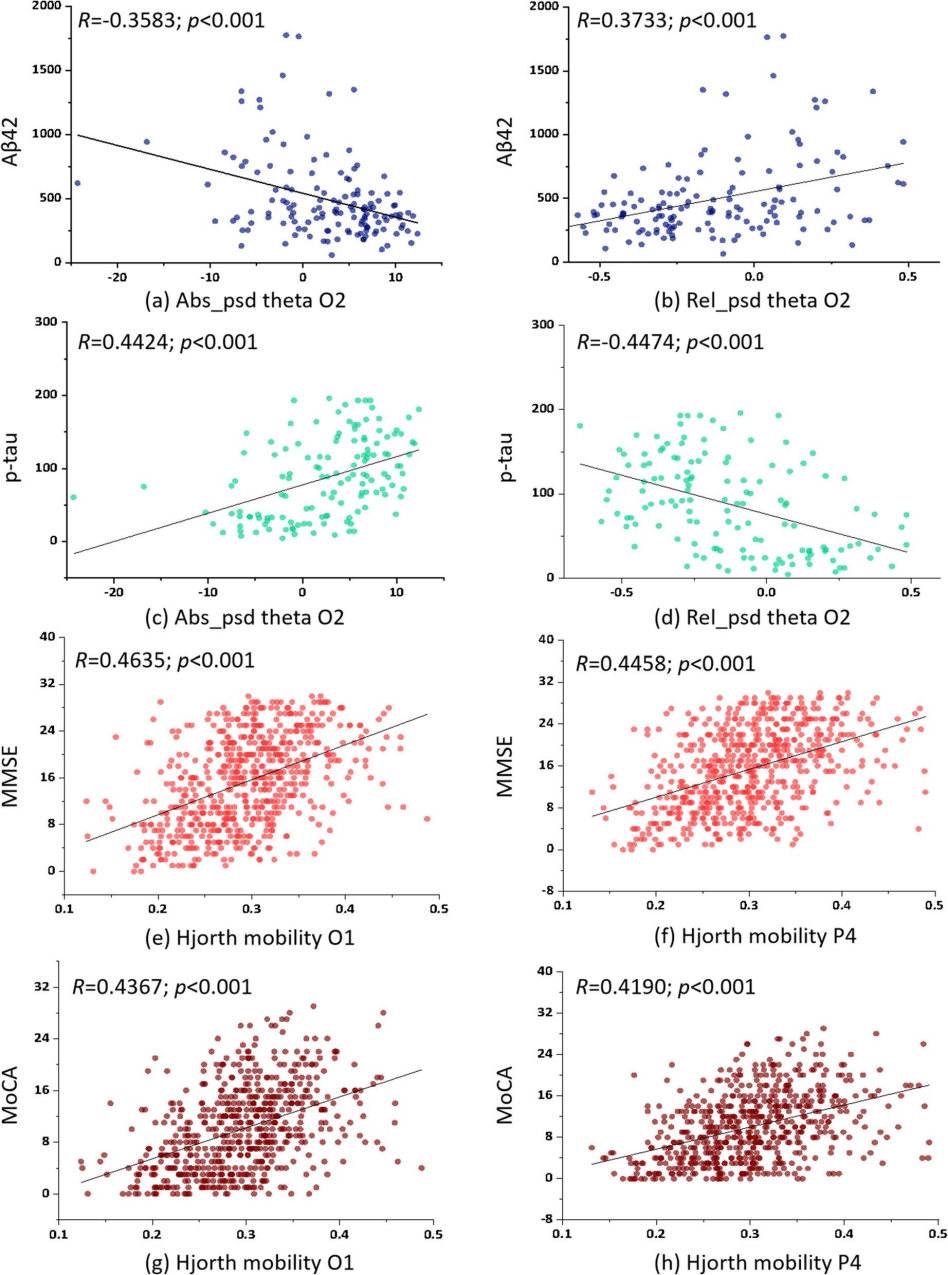
Brain-cognition-CSF relationship in patients with MCI and AD. **a** Absolute theta PSD at O2 vs. Aβ42, **b** relative theta PSD at O2 vs. Aβ42, **c** Absolute theta PSD at O2 vs p-tau, **d** relative theta PSD at O2 vs. p-tau, **e** Hjorth mobility at O1 vs. MMSE, **f** Hjorth mobility at P4 vs. MMSE, **g** Hjorth mobility at O1 vs. MoCA, and **h** Hjorth mobility at P4 vs. MoCA

Results of the correlational analyses among brain measures (EEG features), cognitive decline (MMSE, MoCA), and CSF biomarkers are summarized in Fig. 4. The key EEG features that were effective in distinguishing among HC, MCI, AD, DLB, VCI, and FTD were also significantly correlated with multiple cognition/CSF measures. In particular, the absolute theta power of channel O2 was negatively correlated with Aβ42 (*r* = −0.358, *p* < 0.001) and positively correlated with p-tau (*r* = 0.442, *p* < 0.001), indicating that stronger low-frequency oscillation at the occipital cortex was linked to lower Aβ42 and higher p-tau amount in patients with MCI and AD. Besides, the relative theta power of channel O1 was positively correlated with Aβ42 (*r* = 0.373, *p* < 0.001) and negatively correlated with p-tau (*r* = −0.447, *p* < 0.001). That is, a stronger low-frequency component at the left occipital cortex was associated with a higher amount of Aβ42 and a lower amount of p-tau in the patients. In terms of cognitive assessment, we found Hjorth mobility of channels O1, O2, and P4 were positively associated with MMSE and MoCA scores, with estimated correlations ranging from 0.416 to 0.464 (all *p* values < 0.001) (Fig. 5). This finding suggested that stronger instantaneous fluctuation of EEG signals at the parietal and occipital areas was specifically linked to better cognitive function in patients with cognitive impairment.

### Assessing AD-related behaviors using multimodal features

**Fig. 6:**
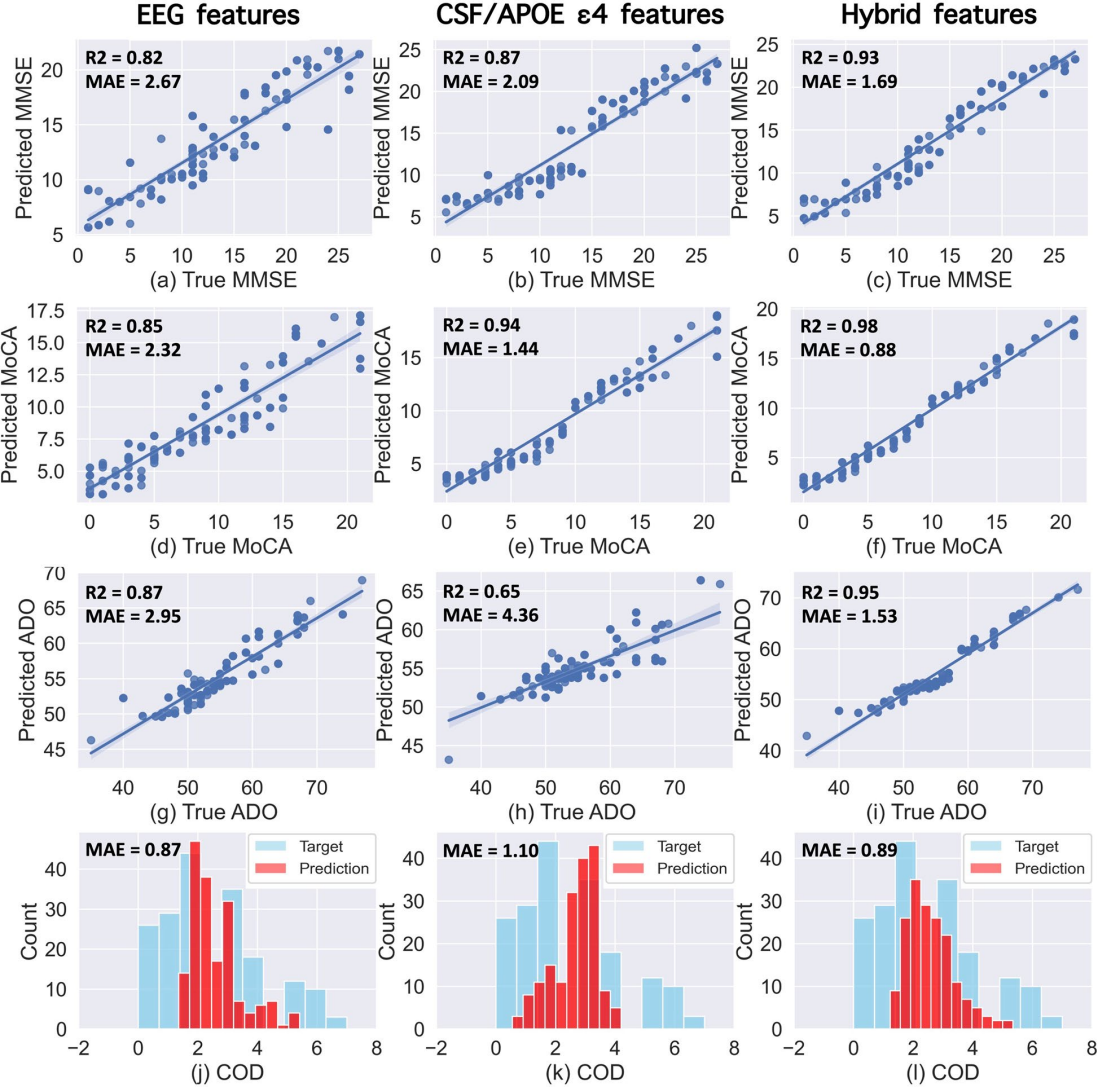
Results of the prediction analyses using different combinations of features. The predictions of MMSE (**a**–**c**), MoCA (**d**–**f**), ADO (**g**–**i**), and COD (**j**–**l**) were obtained using EEG feature only (first column), CSF/*APOE* biomarkers (second column), hybrid features (EEG, CSF/*APOE*, sex, and age) as the predictors of the regression model, respectively. *R*^2^ is the determination coefficient, and MAE is the mean absolute error

The assessment performance of each target measure, including the MMSE score, MoCA score, ADO, and COD, are shown in Fig. 6. For the assessment of the patient’s MMSE score (Fig. 6a–c), EEG-only features achieved moderate performance (MAE = 2.67, *R*^2^ = 0.82), while CSF-*APOE* features achieved slightly better performance (MAE = 2.09, *R*^2^ = 0.87). The best performance was obtained using hybrid features as predictors of the regression model (MAE = 1.69, *R*^2^ = 0.93). Similar results were obtained in the assessment of the patient’s MoCA score (Fig. 6d–f). EEG-only features achieved moderate performance (MAE = 2.32, *R*^2^ = 0.85), whereas CSF-*APOE* biomarkers achieved better performance (MAE = 1.44, *R*^2^ = 0.94). The hybrid features (MAE = 0.88, *R*^2^ = 0.98) were most effective in the assessment of MoCA scores.

In the assessment of ADO (Fig. 6g–i), EEG-only features achieved moderate performance (MAE = 2.95 years, *R*^2^ = 0.87), which was markedly higher compared to the performance obtained from CSF-*APOE* features (MAE = 4.36 years, *R*^2^ = 0.65). The best prediction of ADO was obtained from the hybrid features (MAE = 1.53 years, *R*^2^ = 0.95). For COD (Fig. 6j–l), EEG-only features achieved the best performance (MAE = 0.87 years, no correlation coefficient due to the discrete and small range of COD) compared to a moderate performance using hybrid features (MAE = 0.89 years) or CSF-*APOE* features (MAE = 1.12 years).

## Discussion

Growing evidence has demonstrated that EEG measurements reflect the effects of AD neuropathology on the brain neural signal transmission underpinning cognitive processes [3, 34–36], yet it remains largely unknown about the accuracy and reliability of different types of EEG biomarkers in facilitating the early detection and prediction of AD progression. To our knowledge, this is the first study to comprehensively evaluate the impacts of promising EEG biomarkers on effectively differentiating patients with MCI, AD, and other subtypes of dementias from HC, and to clarify the associations among EEG-based neural signatures, individual cognitive function, and CSF biomarkers. Remarkably, we provided strong evidence supporting that the inclusion of multi-dimensional information (i.e., EEG biomarkers, CSF/*APOE* ε4 measures, and demographic characteristics) is highly effective in assessing patients’ cognitive function and clinical status through a machine learning approach. Together, our findings suggest that EEG biomarkers are of great importance for the early detection of AD in clinical practice.

Identifying effective EEG biomarkers associated with AD could help us elucidate the neural mechanism underlying this neurodegenerative disorder and facilitate its early diagnosis. As reported in previous studies, patients with MCI and AD generally showed decreased alpha/beta power and increased theta/delta power in a broad range of brain regions such as frontal, temporal, parietal, and occipital areas [23, 37–39]. Other abnormal brain alterations, such as functional connectivity and entropy, were also reported [17, 37, 40–42]. However, the multi-group classification of HC, MCI, and AD based on these EEG biomarkers has not yet achieved satisfactory accuracy, possibly due to the difficulty in selecting meaningful EEG metrics and the heterogeneity of patients when the sample size is limited. In this study, we addressed this challenge by recruiting a relatively large sample of healthy controls (246) and patients (189 MCI and 330 AD) in a clinical framework and achieved a decent three-level classification accuracy (over 70%). In particular, the key EEG biomarkers that have the most prominent effect on differentiating MCI and AD from the HC included power spectral density measures and entropy, which aligns well with previous studies [37, 43]. Our findings thus confirm that the power spectral density metrics and entropy are significantly altered in AD patients. Importantly, given the decent sample size and the rigorous EEG acquisition condition in the present study, our findings could provide a solid benchmark for developing useful EEG-based tools for the early diagnosis and screening of AD.

We explored whether EEG-based biomarkers could differentiate MCI and AD from other subtypes of dementia, a critical clinical question that has never been systematically studied before. We found that multiple EEG biomarkers revealed highly distinct patterns over MCI/AD-related groups and other dementias. First, the spectral measures of theta rhythm (absolute/relative PSD) at the occipital region presented with a purely monotonic trend from HC to MCI and AD, a finding that has been consistently reported in previous studies [14, 37]. Post hoc pair-wise comparisons further confirmed that these measures were capable of differentiating HC/MCI/AD groups from other dementias. We hypothesize that the alterations of the EEG power spectral density may be present not only in AD-related cognitive decline, but also in other dementias that have different neurodegenerative causes (e.g., FTD, VCI). And such alterations could be potentially differentiated by EEG measurements. This premise is supported by recent studies suggesting that EEG technique may be able to distinguish MCI/AD patients from other dementias such as FTD, VCI, or DLB [18–20]. However, it is worth noting that we didn’t perform a systematic classification among all six groups in this study due to the relatively small sample sizes of FTD, VCI, and DLB groups. Future work is needed to evaluate the effectiveness of EEG techniques in diagnosing different types of dementias.

We found that Hjorth mobility of EEG signals at parietal and occipital regions was highly effective in distinguishing among HC, MCI, AD, DLB, VCI, and FTD groups. This was evidenced by the consistently decreased mobility in the HC-MCI-AD trajectory and the significant differences in most pair-wise comparisons among all six groups. Hjorth mobility is one of the Hjorth parameters that measure the complexity of a signal [44]. Among the Hjorth parameters, mobility is defined as a ratio per time unit and may also be treated as a mean frequency. Though less explored in AD studies, decreased Hjorth complexity was reported in the AD cohort compared to HC [45]. Previous studies also showed that the complexity measure of EEG could be effective in the differential diagnosis of MCI and AD [24, 46], which strengthens the findings in our study. Besides, the decreased mobility in MCI and AD group reflects the decreased frequency of neuronal oscillation caused by cognitive decline. This finding is in line with a previous study that reported a shift in the power spectrum toward the lower frequencies (delta and theta band) [37]. Given the high discrimination power of these biomarkers that have never been reported in the literature, our study provides first-hand evidence for developing EEG-based quantitative, statistically significant criteria that could be applied to the differential diagnosis of dementia in routine assessments in the future.

Understanding the relationship between EEG biomarkers and currently used biomarkers (e.g., CSF) could improve our knowledge of the pathological basis of AD. With a large sample size, we found that key EEG biomarkers, including power spectrum at the occipital region and signal complexity at the parieto-occipital area, were significantly associated with both patient’s cognitive decline and levels of p-tau and Aβ42. These findings consolidate results from previous studies that reported significant correlations between EEG-derived biomarkers and CSF measures (e.g., Aβ42, tau, p-tau) in limited samples of AD patients [47, 48]. Together with the above classification result, in addition to being able to detect AD and other dementias, EEG biomarkers may be used as potential measures for monitoring the progression of AD. In particular, CSF and neuroimaging biomarkers have been a core basis in recently proposed recommendations and research criteria for the diagnosis of AD at the preclinical, prodromal, and overt dementia stages in the clinical practice [4, 49]. Therefore, EEG-derived biomarkers could be a valuable addition to the clinically adopted neuroimaging biomarkers.

To further evaluate the added value of including EEG in clinical practice, we implemented a machine learning approach to assess patients’ cognitive decline, ADO, and COD using standalone EEG biomarkers and a combination of EEG and other recommended biomarkers. CSF/*APOEε4* measures were more effective than EEG biomarkers in assessing patients’ cognitive function (i.e., MMSE and MoCA). This is expected given that ATN measures have been widely accepted as recommended biomarkers for clinical diagnosis of AD [4, 50]. Yet, EEG biomarkers were found to be more effective than the CSF/*APOE* measures in assessing disease ADO and COD, suggesting the advantage of using EEG to evaluate the temporal profile of AD. As expected, the combination of the measures of EEG, CSF, *APOE ε4*, age, and sex achieved the best performance in all assessments. It has been recommended that the combination of amyloid-based biomarkers with other measures such as abnormal neurodegeneration biomarkers could provide higher accuracy and reliability in the prediction of future cognitive decline and conversion rate to AD than amyloid measure alon e[51]. Our finding thus supports the premise that the fusion of multi-dimensional information could potentially improve the power of primary outcomes in clinical trials.

Several limitations of this study should be acknowledged. First, we have a limited sample size for other subtypes of dementia, such as FTD, VCI, and DLB. Though the focus of this study is to perform a three-way classification of HC/MCI/AD, future work should include various types of dementia to assess the sensitivity and specificity of EEG-based early detection of AD. In addition, due to the limited availability of CSF/*APOE* measures, only a small number of MCI and AD patients are available for the prediction analysis in the present study. The effectiveness of adding EEG biomarkers into a model for monitoring and prediction of AD progression should be evaluated with larger samples in the future. Finally, this study only included measurements for a single time point. Longitudinal studies with large cohorts would be conducted in the future to assess whether EEG biomarkers can be used to trace the progress trajectory of AD or evaluate the efficacy of pharmacological/therapeutic interventions for AD patients.

## Conclusions

Increasing evidence suggests that EEG biomarkers are diagnostically meaningful and associated with the clinical progression of AD. In this study, we identified distinct neural biomarkers that were specifically linked to the CSF measures and cognitive function of AD patients. These neural biomarkers mainly included the power spectrum alterations of low-frequency oscillations at the occipital area and the altered signal complexity at the parietal and occipital regions. Finally, through a machine learning approach, we found that the combination of EEG biomarkers, CSF/*APOE ε4* measures, and demographic information of patients was most effective in assessing individual cognitive function and disease progression.

## Supplementary Information

**Additional file 1.** Supplementary material.

## Data Availability

The datasets generated and/or analyzed in the present study are available from the corresponding author upon reasonable request.

## Abbreviations

AD: Alzheimer’s disease
ADO: Age of disease onset
ATN: Amyloid/Tau/Neurodegeneration
COD: Course of disease
CSF: Cerebrospinal fluid
DLB: Dementia with Lewy bodies
EEG: Electroencephalogram
FTD: Frontotemporal dementia
HC: Healthy controls
LDA: Linear discriminant analysis
MAE: Mean absolute error
MCI: Mild cognitive impairment
MMSE: Mini-Mental State Examination
MoCA: Montreal Cognitive Assessment
PET: Positron emission tomography
SVM: Support vector machine
VCI: Vascular cognitive impairment
