# Supplementary material for: Neural biomarker diagnosis and prediction to mild cognitive impairment and Alzheimer’s disease using EEG technology
Source: Alzheimers Res Ther. 2023 Feb 10;15:32. doi: 10.1186/s13195-023-01181-1 (PMC9912534; doi:10.1186/s13195-023-01181-1)
Supplement: Supplementary file 1 — Additional file 1. Supplementary material. [file 13195_2023_1181_MOESM1_ESM.docx]

**Neural biomarker diagnosis and prediction to mild cognitive impairment and Alzheimer's disease using EEG technology**

We extracted multiple types of EEG features that have been widely reported in previous studies. These features included absolute power spectrum density, relative power spectrum density, Hjorth metrics (activity, mobility, and complexity), sample entropy, time-frequency property (STFT), and microstate measures (lifetime, occurrence rate, converting rate). Definitions of these features are described in detail below.

1. **Absolute/Relative Power Spectral Density (PSD)**

Power spectral density (PSD) is a well-established metric for the analysis of EEG signals. PSD usually represents the power distribution of EEG series in the frequency domain. In this study, we first used the most widely used Welch's periodogram method to compute the PSD of the EEG time series for each frequency band and each channel [1]. The resulting PSD is the absolute PSD of the EEG at a specific frequency band and channel. The relative PSD is simply defined as the ratio of the PSD of a specific frequency band to the total frequency band. The absolute PSD and relative PSD features were extracted from each frequency band of each channel.

1. **Hjorth Metrics**

Hjorth parameters are variance features representing the derivation of the EEG signals. Among that, the first three derivation Hjorth parameters of the brain signals are activity, mobility, and complexity, and there are critical analytical characteristics in this work [2]. The related computation formulas are presented as follows:

1. The Hjorth activity parameter represents the signal power and the variance of a time series, and further indicates the surface of the power spectrum in the frequency domain. The Hjorth activity can be calculated by the following formula:

$Activity=var(y(t))$ (1)

where *y*(*t*) is the EEG time series of any channel and var (.) is the variance operation.

1. The Hjorth mobility parameter represents the mean frequency or the proportion of standard deviation of the power spectrum. Hjorth mobility is defined as the square root of the variance of the first derivative of the signal *y*(*t*) divided by variance of the signal *y*(*t*), given as:

$Mobility=\sqrt{\frac{var(\frac{dy(t)}{dt})}{var(y\left( t \right))}}$ (2)

1. The Hjorth complexity parameter represents the change in the frequency of a signal. The parameter compares the signal's similarity to a pure [sine wave](https://wikimili.com/en/Sine_wave), where the value converges to 1 if the signal is more similar. The *complexity* of the EEG at any channel is given by:

$Complexity=\frac{Mobility(dy(t)/dt)}{Mobility(y(t)}$ (3)

The Hjorth metrics were computed for each channel.

1. **Sample Entropy**

Sample entropy (SampEn) is a novel and improved metric to assess the complexity and certain self-similarity of a time series [3]. The function of SampEn, listed below, is the negative of logarithmic that two similar sequenced of *m* consecutive data points remain similar at the next point *(m+1)* or not:

$hq(m,r)=log(\frac{Cq(m,r)}{Cq(m+1,r)})$ (4)

where *m* is the embedding dimension and *r* is the radius of the neighborhood. The detailed mathematical calculation of SampEn can be found in [4]. Note that the SampEn *hq*(*m*,*r*) should not change for both various *m* and *r*. In this study, we calculated the SampEn for each channel using the parameter m = 2 and r = 0.1.

1. **Time-frequency property based on Short-Time Fourier Transform**

The Short-Time Fourier Transform (STFT) is used to analyze how the frequency content of a signal changes over time. Specifically, STFT is to divide a longer-time signal into shorter segments of the same length and computes the Fourier transform on each shorter segment. The equation is defined as follows:

$STFT\left\{ x(t) \right\}\left( \tau,\omega\right)=X\left( \tau,\omega\right)=\int_{-\infty}^{\infty} x(t)\omega(t-\tau)e^{-ı\omega t}dt$ (5)

where *ɷ(τ)* is the window function, commonly a Hann window or Gaussian window centered around zero. *x(t)* is the EEG signal to be transformed. *X(τ,ω)* is essentially the Fourier Transform of *x(t)ɷ(t−τ)*, a complex function representing the phase and magnitude of the signal over time and frequency. In this study, the entropy, kurtosis, skewness, mean, and standard deviation of the STFT of each frequency band and each channel were computed.

1. **Microstate Measures**

EEG Microstate explains the states as the topographies of electric potential over all the EEG electrodes, essentially a view of the spatial distribution of the electric potential on the scalp at each time point [5]. Global field power (GFP) is computed to obtain EEG microstates in the resting state or spontaneous EEG, and it is simply given as the root mean square across average-referenced electrode values at a given time instant. The formula is defined as follows:

$GFP=\left[ \sum_{n=1}^{M} {(V_{n}(t)-\hat{V}(t))}^{2})/N \right]^{1/2}$ (6)

*GFP* is the spatial standard distribution of the brain signal, where *Vn(t)* is the electric potential at *t* and *n*th electrode, and $\hat{V}(t)$ is the average electric potential across all M electrodes at *t*. We then computed three measures of the EEG microstates across all channels, including the average length of time a given microstate remains stable whenever it appears (lifetime), the average number of times per second that the microstate becomes dominant during the recording period (occurrence frequency), and the transition probabilities of a given microstate to any other microstate (converting rate).

**References:**

1. Welch, Peter. "The use of fast Fourier transform for the estimation of power spectra: a method based on time averaging over short, modified periodograms." IEEE Transactions on audio and electroacoustics 15.2 (1967): 70-73.
2. Hjorth, B., 1970. EEG analysis based on time domain properties. Electroencephalogr. Clin. Neurophysiol. 29, 306-310.
3. Delgado-Bonal, A., Marshak, A., 2019. Approximate entropy and sample entropy: a comprehensive tutorial. Entropy 21, 541.
4. J.-R. Huang, S.-Z. Fan, M. F. Abbod, K.-K. Jen, J.-F. Wu, and J.-S. Shieh, “Application of multivariate empirical mode decomposition and sample entropy in EEG signals via artificial neural networks for interpreting depth of anesthesia,” Entropy, vol. 15, no. 9, pp. 3325–3339, 2013.
5. Koenig T, Prichep L, Lehmann D, Sosa PV, Braeker E, et al. (2002) Millisecond by millisecond, year by year: normative EEG microstates and developmental stages. Neuroimage 16: 41–48.
